# Acousto-Electric Conversion Fiber Networks via Regional Activation of Schwann Cell-Derived Exosomes for Neurogenic Bone Regeneration

**DOI:** 10.34133/research.0769

**Published:** 2025-07-15

**Authors:** Weiwei Yi, Xiaoyu Han, Fan Wang, Qiuyu Tang, Huzhe Liu, Bo Liao, Jieliang Shen, Juan Wang, Wenguo Cui, Dingqun Bai

**Affiliations:** ^1^ Department of Rehabilitation Medicine, Key Laboratory of Physical Medicine and Precision Rehabilitation of Chongqing Municipal Health Commission, The First Affiliated Hospital of Chongqing Medical University, Chongqing 400016, China.; ^2^ Department of Orthopaedics, Shanghai Key Laboratory for Prevention and Treatment of Bone and Joint Diseases, Shanghai Institute of Traumatology and Orthopaedics Ruijin Hospital Shanghai Jiao Tong University School of Medicine, Shanghai 200025, China.; ^3^ Department of Orthopaedics, People’s Hospital of Chongqing Liang Jiang New Area, Chongqing 400016, China.; ^4^ Department of Rehabilitation Medicine, Bishan Hospital of Chongqing Medical University, Bishan Hospital of Chongqing, Chongqing 402760, China.

## Abstract

Neurogenic bone regeneration is essential for the effective restoration of bone tissue functionality, with exosomes derived from Schwann cells regionalized in bone injury tissue playing a crucial role in this process. However, precisely regulating the secretion of Schwann cells localized in bone injury tissue to enhance neurogenic bone regeneration remains a considerable challenge. In this study, an injectable, ultrasound-responsive piezoelectric conductive short fiber network (US@SFG) was innovatively developed using uniform short fiber homogenization techniques and multifunctional chemical modifications, enabling precise acoustic–electrical conversion that regionally activated the secretion of miRNAs from Schwann cell-derived exosomes, thereby promoting neurogenic bone regeneration. The incorporation of the piezoelectric polymer glycine imparts superior piezoelectric characteristics to the fiber network, while the conjugated π-electron motion within the conductive graphene network enhances internal electron transfer efficiency, thereby facilitating electrical conductivity. Compared with traditional piezoelectric fiber networks, acousto-electric conversion fiber networks demonstrated a 1.7-fold increase in piezoelectric performance and a 30-fold increase in conductivity, facilitating precise electrochemical regulation under ultrasound stimulation. In vitro studies revealed that acousto-electric conversion fiber networks precisely modulate the secretion of localized Schwann cell exosomal miRNAs (miRNA-494-3p, miRNA-381-3p, and miRNA-369-3p), activating the phosphatidylinositol 3-kinase/protein kinase B and Wnt signaling pathways in bone marrow mesenchymal stem cells, and thereby promoting osteogenic differentiation. Furthermore, in vivo experiments confirmed that under ultrasound imaging guidance, acousto–electric conversion fiber networks could be directed precisely to bone defects, where precise control of ultrasound parameters facilitated acoustic–electrical conversion and electrical signal modulation, markedly promoting the formation of neural networks and bone tissue regeneration. In this study, for the first time, an injectable acousto-electric conversion fiber network was constructed to activate Schwann cell exosomes in bone injury tissue regionally, providing a novel therapeutic strategy and potential molecular targets for neurogenic bone regeneration.

## Introduction

Fractures represent an important global health challenge, with an estimated annual incidence of approximately 620 million cases worldwide. Although bone tissue possesses an inherent regenerative capacity, the fracture healing process markedly impacts patients’ functional outcomes and quality of life. Epidemiological studies indicate that 10% to 20% of fractures result in compromised healing outcomes, such as delayed union or nonunion, which continue to pose substantial clinical concerns. The scarcity of effective treatment options underscores the urgent necessity to explore the molecular and cellular mechanisms governing bone repair, potentially paving the way for innovative therapeutic strategies to enhance fracture healing.

Bone, as a highly innervated and sophisticated organ, undergoes functional reconstruction that is intrinsically linked to neural regeneration and functional recovery [1]. Schwann cells (SCs), the most abundant and primary glial cells in the peripheral nervous system, are essential for maintaining the neural innervation of both the peripheral nervous system and newly formed bone [2,3]. Upon bone injury, peripheral nerves are damaged, leading to the proliferation of SCs, which secrete a variety of neurotrophic factors or exosomes in a paracrine manner to support local nerve regeneration and bone repair [4,5]. However, current research has focused primarily on loading SCs or their exosomes onto biomaterials for delivery to bone injury sites to promote regeneration and repair [6,7]. However, several challenges remain: (a) how to ensure the optimal release and activity of SCs and their secreted exosomes around injured bone tissue; (b) the potential impact of SC proliferation and differentiation on their phenotype and function, which may influence the consistency and predictability of treatment outcomes; and (c) how to design biomaterials that ensure the long-term stability of regionalized activated SCs and their exosomes in vivo. Therefore, developing therapeutic strategies that precisely regulate SC exosomes at bone injury sites to effectively promote neurotrophic bone repair could lead to improved bone tissue regeneration and reconstruction outcomes.

Bone tissue, as an electrically sensitive structure, is highly responsive to electrical signals, which play crucial roles in both physiological bone activity and injury repair [8–10]. Similarly, SCs—which are also electrically excitable—respond to electrical stimulation by increasing their proliferation and the secretion of various cytokines, thereby accelerating nerve regeneration and bone repair processes [11,12]. To recapitulate endogenous electric fields in bone tissue for enhanced regeneration, researchers have developed functional electrodes that deliver exogenous electrical stimulation or utilize piezoelectric materials to promote SC migration and proliferation [13–16]. However, current approaches face marked limitations: external electrical stimulation requires complex operation with poor spatial and intensity control, and piezoelectric materials merely generate electricity without precise signal modulation or effective conduction, ultimately failing to restore physiological electrical signaling in damaged bone tissue. Therefore, developing materials that can amplify the electrical signals generated by the piezoelectric effect and improve the signal conduction performance of piezoelectric materials is crucial for enhancing the synchronous repair of neural and bone tissues.

During the process of bone repair, the generation and conduction of electrical signals primarily rely on the dynamic mechanical stresses of tissues. However, patients with bone injuries often require prolonged immobilization, which limits the ability to generate effective electrical stimulation through the dynamic mechanical stresses of tissues. Additionally, the magnitude of these dynamic stresses in tissues is uncontrollable. Consequently, to efficiently and controllably harness the bone repair potential of piezoelectric conductive materials, a noninvasive and controllable external driving method must be identified. Ultrasound is a noninvasive, precise, and highly penetrative physical therapy modality with the added advantage of imaging capability [17,18]. Ultrasound imaging enables the precise injection of electroactive materials into the fracture site while simultaneously serving as a tool for fracture diagnosis and treatment evaluation. Compared with x-ray and computed tomography (CT), ultrasound has the advantages of being radiation-free and safer for diagnosis. Moreover, ultrasound can deliver continuous mechanical stimuli to electroactive materials, providing sustained, precise electrical stimulation to the fracture site. By controlling the ultrasound intensity, the strength of the electrical signals within the body can be regulated. Therefore, the development of ultrasound-responsive electroactive materials has the potential to enable precise control of ultrasound parameters, facilitating efficient acoustic–electrical conversion and signal modulation, thereby markedly promoting the formation of regionalized neural networks and bone tissue regeneration.

**Fig. 1.**
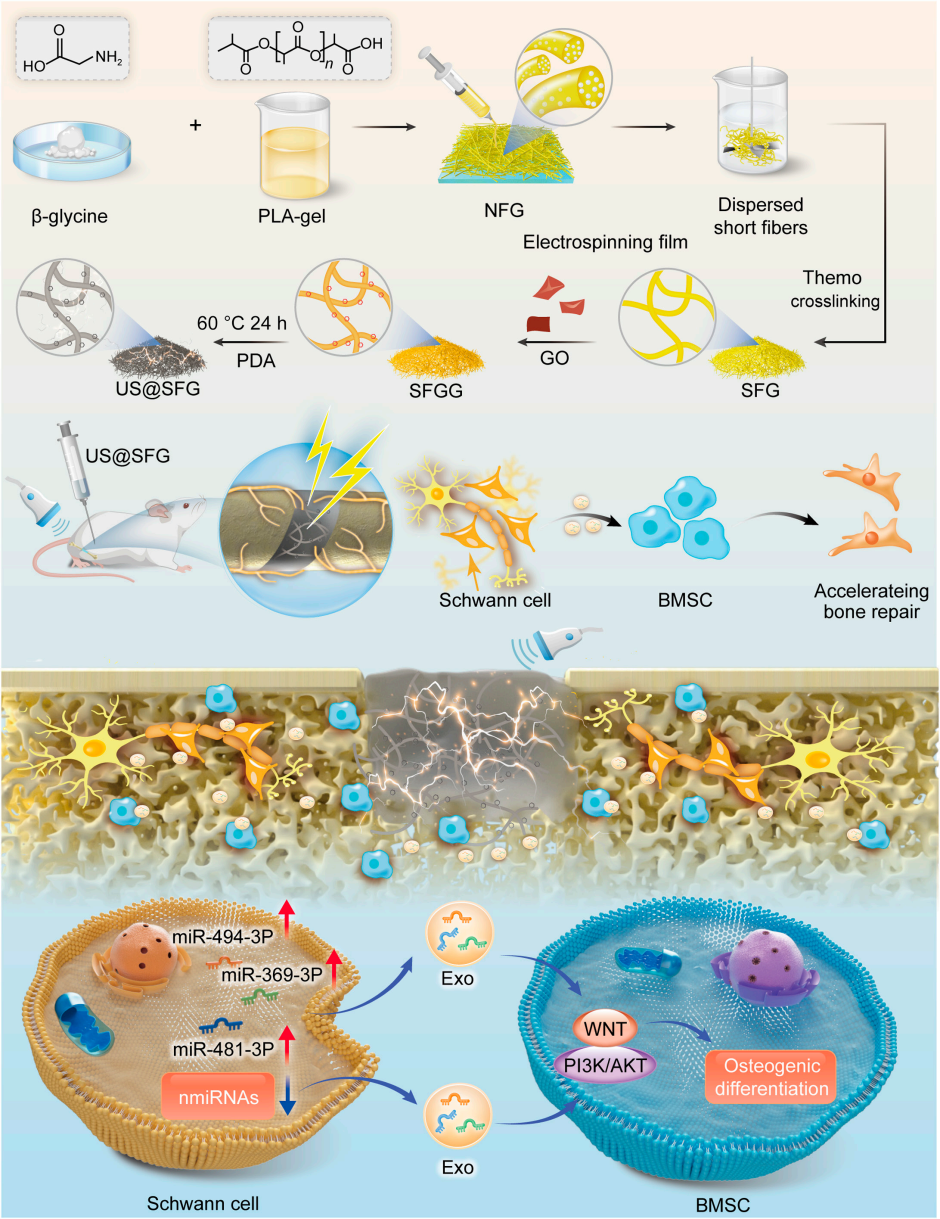
The design of an acousto-electric conversion fiber network (US@SFG) and the mechanism of regional activation of Schwann cell-derived exosomes in promoting neurogenic bone repair.

This study represents a novel advancement in the development of injectable acousto-electric acoustic and electric conversion fiber networks that activate bone marrow stromal cells (BMSCs) by regulating the secretion of exosomal miRNA-494-3p, miRNA-381-3p, and miRNA-369-3p from SCs in the region of bone injury, thereby promoting bone-related phosphatidylinositol 3-kinase/protein kinase B (PI3K/Akt)and Wnt signaling pathways and ultimately accelerating neurogenic bone repair (Fig. 1). Initially, piezoelectric glycine/polylactic acid (PLA)/gelatin short fiber networks were fabricated using electrospinning and fiber homogenization techniques. Subsequently, graphene oxide (GO) was uniformly grafted onto piezoelectric short fiber networks through π–π conjugation, and mussel-inspired polydopamine (PDA) was used to effectively induce redox reactions in GO, thus creating injectable ultrasound-responsive piezoelectric-conductive short fiber networks (US@SFG). These acousto-electric conversion fiber networks can be precisely injected into bone injury sites under ultrasound imaging guidance. Mechanical stimulation from ultrasound waves enables precise acoustic–electrical conversion within the tissue. Furthermore, controlling the ultrasound parameters allows for fine-tuning of the electrical signal intensity. In vitro, after coculturing SCs with US@SFG, exosomes were extracted from the supernatant, and their effects on the osteogenic differentiation of BMSCs were evaluated. High-throughput exosome sequencing and bioinformatics analysis were performed to predict the osteogenic miRNAs and related target gene pathways in the exosomes. In vivo, a rat femoral fracture model was established, and imaging and histological analyses were used to validate the role of US@SFG in promoting neural and bone repair. Finally, transcriptomic analysis was employed to further elucidate the mechanisms of the localized activation of SC exosomes in neuro-bone repair by acousto-electric conversion fiber networks. In conclusion, the injectable acousto-electric conversion fiber network developed in this study offers a novel therapeutic approach and potential targets for the interaction between bone and innervating neurons.

## Results and Discussion

### Preparation and characterization of acousto-electric conversion fiber networks

**Fig. 2.**
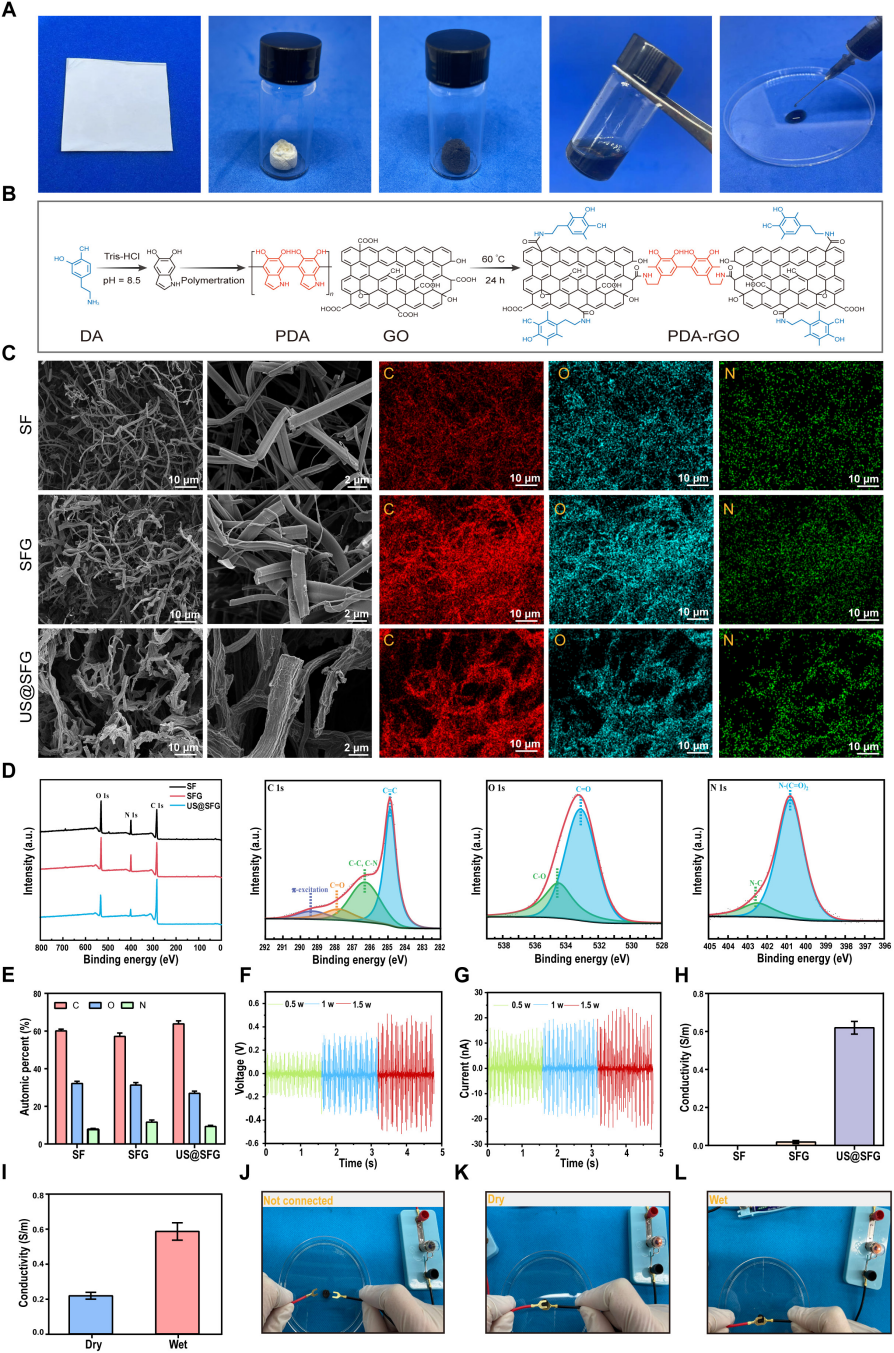
Preparation and characterization of acousto-electric conversion fiber networks: (A) Macroscopic images of the short fiber networks in different states. (B) Schematic of the reduction mechanism of graphene oxide (GO) by dopamine (DA). (C) SEM images of different types of short fiber networks at various magnifications. (D) Representative XPS spectra of different short fiber networks. (E) Elemental composition (C, O, and N) in the different short fiber networks, expressed as atomic percentages. (F and G) Piezoelectric properties of the electroactive short fiber networks under different ultrasound intensities. (H) Conductivity of the acousto-electric conversion fiber networks. (I) Conductivity of the acousto-electric conversion fiber networks (US@SFG) under dry and wet conditions. (J to L) Conductivity of the acousto-electric conversion fiber networks (US@SFG) in integrated circuits under dry and wet conditions.

An ultrasound-activated piezoelectric conductive integrated injectable short fiber network was prepared as shown in Fig. 2. Initially, the short fiber networks were white, 2-dimensional fiber membranes with random orientations. After homogenization, the long fibers within the membrane were transformed into short fibers. Following freeze-drying, the material became a powder composed of 3-dimensional short fiber networks (SF) and mixed glycine short fiber networks (SFG). The materials then underwent thermal crosslinking, followed by GO modification and the reduction of GO by PDA. Owing to its redox activity and stability, PDA is more effective than conventional reductants in obtaining conductive reduced graphene (rG) [19]. The synthesized SFG-GO is submerged in a basic dopamine (DA) solution, where DA undergoes self-polymerization to form PDA. Subsequently, PDA is polymerized onto the SFG-GO scaffold, facilitating the in situ reduction of GO to rG through amide bond formation (Fig. 2B). As shown in Fig. 2A, the initially white crosslinked SFG gradually turned brown and eventually blackened, forming US@SFG. This color change can be attributed to the intrinsic brownish-yellow hue of the GO dispersion, which coated the nanofibers and imparted a brownish tint. Subsequent reduction with PDA converted GO into black rGO, confirming the successful modification of rGO onto the short fibers. Scanning electron microscopy (SEM) revealed abundant fragmented short fibers, confirming the successful preparation of electrospun fiber membranes into short fibers with smooth surfaces. Compared with SFG, US@SFG, which consists of rGO-coated SFG fibers, exhibited distinct curling and wrinkling (Fig. 2C), indicating successful PDA coating. Notably, neither PDA nor rGO disrupted the fibrous network’s biomimetic structure. To further validate the successful incorporation of the β-glycine and rGO modifications into the short fibers, we performed x-ray photoelectron spectroscopy (XPS) analysis and evaluated their piezoelectric and conductive properties. As shown in Fig. 2D and E, all 3 fiber groups primarily contained the elements C, N, and O. Compared with the SFG group, the SFG group presented increased N content, whereas the US@SFG group presented decreased O content. This is attributed to N being predominantly present in glycine, whereas the rGO coating masked some surface elements of the fibers. The piezoelectric properties of US@SFG fibers were evaluated under ultrasound stimulation at various intensities (0.5, 1.0, and 1.5 W/cm^2^). As shown in Fig. 1F and G, US@SFG demonstrated approximately 1.7-fold higher piezoelectric output (current and voltage) compared to SFG fibers (Fig. S2). Electrical conductivity measurements revealed that although pure SF showed undetectable conductivity and SFG exhibited minimal conductivity, US@SFG displayed a remarkable 30-fold increase in conductivity versus SFG, confirming successful rGO modification (Fig. 2H). The conductivity of US@SFG was further tested under both dry and wet conditions (immersed in 0.9% saline buffer) (Fig. 2I). The conductivity of the fiber networks was notably greater under wet conditions than under dry conditions. Finally, integrated circuit tests revealed that the brightness of a light-emitting diode under wet conditions was greater than its brightness under dry conditions (Fig. 2J to L).

### Biocompatibility of acousto-electric conversion fiber networks

**Fig. 3.**
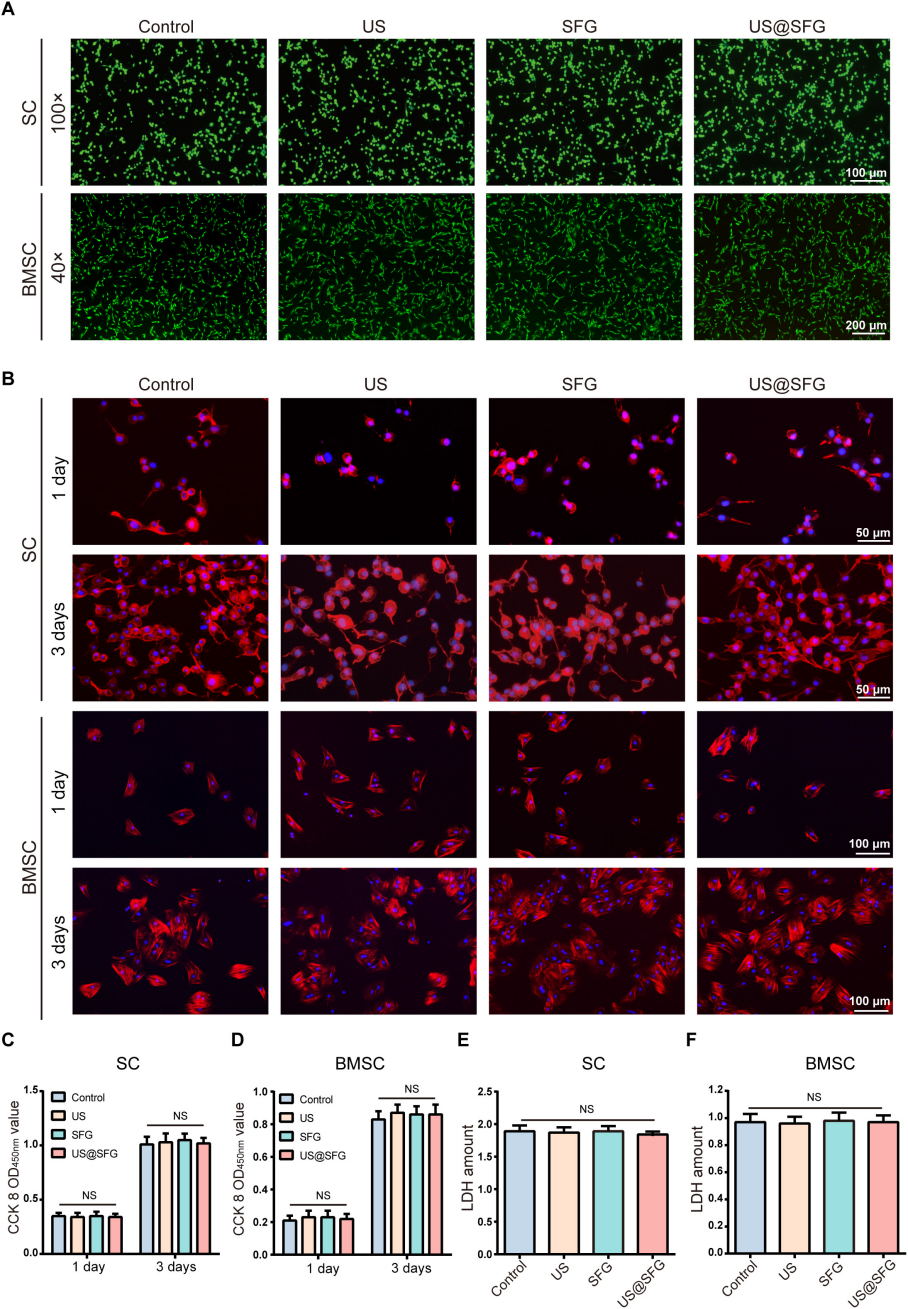
Biocompatibility of acousto-electric conversion fiber networks: (A) Live/dead staining of cells (SC and BMSCs) cocultured with different samples. (B) Cytoskeletal staining of SCs and BMSCs cocultured with different materials for 1 and 3 days. (C and D) CCK-8 assay measuring cell viability of SCs and BMSCs cocultured with different materials for 1 and 3 days. (E and F) LDH assay measuring cytotoxicity of SCs and BMSCs cocultured with different fiber networks for 24 h (data are reported as mean ± SD; NS, no significant difference).

Biocompatibility represents a fundamental prerequisite for the design of biomaterials for neurogenic bone regeneration. Previous studies have established that this reparative process critically depends on the osteogenic differentiation capacity of BMSCs and the proliferative/secretory functions of SCs [20,21]. We therefore systematically evaluated the biological effects of the US, SFG, and US@SFG materials on these key cell types through comprehensive assessments of their cellular morphology, viability, proliferation, and cytotoxicity. First, the live/dead staining results revealed that the SCs and BMSCs in the 4 groups exhibited good cell morphology and viability. Although a small number of dead cells were observed, the overall proliferation rate remained normal (Fig. 3A). Next, cytoskeletal staining of cocultured cells revealed a rounded morphology on day 1, with well-extended cellular shapes observed by day 3 across all groups (Fig. 3B). Cell Counting Kit-8 (CCK-8) assays revealed normal proliferation rates for both SCs and BMSCs under all experimental conditions. Lactate dehydrogenase (LDH) assays confirmed the low cytotoxicity of the acousto-electric fiber network, meeting standard biocompatibility requirements. These collective findings indicate the excellent biocompatibility of the material (Fig. 3C to F), providing crucial preclinical evidence for future diagnostic and therapeutic development.

### Characterization and internalization of exosomes

**Fig. 4.**
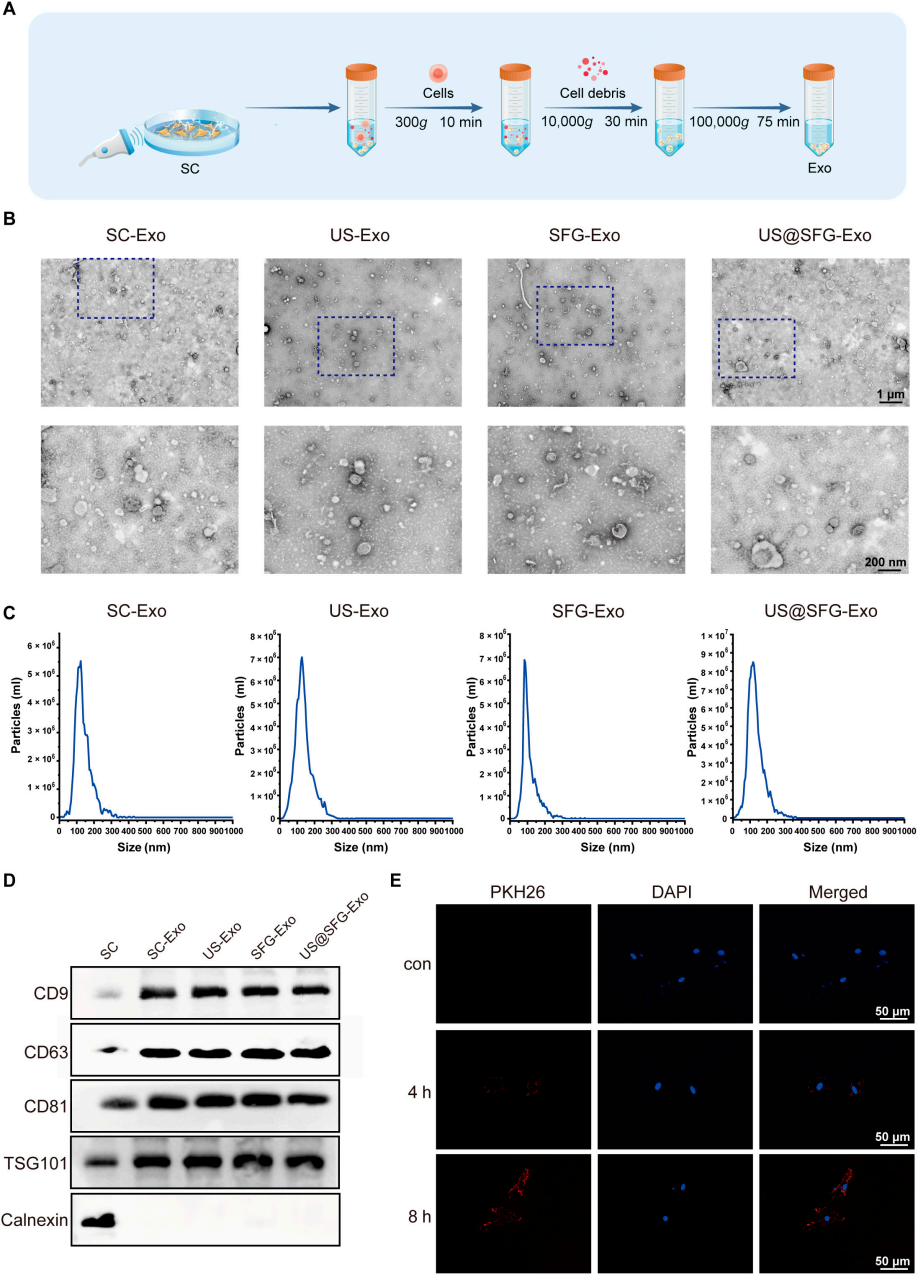
Characterization and internalization of exosomes: (A) Schematic of exosome isolation and purification using differential ultracentrifugation. (B) Transmission electron microscopy (TEM) images showing the morphology of exosomes. (C) Nanoparticle tracking analysis (NTA) of exosome size distribution. (D) Western blotting detection of exosome proteins CD9, CD63, CD81, TSG101, and Calnexin. (E) PKH26-labeled US@SFG-treated SC-derived exosomes uptake by BMSCs.

Current evidence suggests that moderate ultrasound stimulation may promote cell proliferation through the extracellular signal-regulated kinase (ERK) and PI3K-Akt signaling pathways [22]. However, excessive intensity may induce cytoskeletal reorganization or generate detrimental reactive oxygen species levels, ultimately impairing cellular proliferation [23,24]. The CCK-8 assay results indicated that when the ultrasonic stimulation intensity was 1 W/cm^2^, the acousto-electric conversion fiber network (US@SFG) was the most optimal for SC growth and proliferation (Fig. S3). Therefore, the ultrasonic intensity was selected as 1 W/cm^2^ in the subsequent studies. To explore how SC-derived exosomes influence the osteogenic differentiation of BMSCs, exosomes were extracted from the supernatants of SCs cultured for 48 h with US, SFG, or US@SFG, as shown in Fig. 4A. Exosomes were then purified from the supernatant of each group, with exosomes extracted from the culture medium of SCs serving as the blank control group. The exosomes from all 4 groups were characterized and quantified. The exosomes from all the groups displayed similar cup-shaped or spherical morphologies, as revealed by the transmission electron microscopy (TEM) images (Fig. 4B). As shown in Fig. 4C, the particle sizes of the exosomes from all 4 groups were primarily in the range of 80 to 160 nm. Western blot analysis further validated the presence of exosome-specific markers (CD9, CD81, TSG101, and CD63) across all the exosomal preparations, while the negative marker Calnexin was not detected (Fig. 4D). To investigate exosome uptake by BMSCs, PKH26-labeled exosomes were cocultured with BMSCs. Fluorescence microscopy revealed rapid exosome internalization within 4 h, with the signal intensity further increasing by 8 h, demonstrating time-dependent uptake kinetics (Fig. 4E). This result is consistent with the phenomenon that mesenchymal stem cells take up exosomes through an active endocytosis mechanism. Notably, although the morphology or size of the exosomes from the different treatment groups did not differ markedly, their cargo composition (such as that of proteins and miRNAs) may exhibit functional variations, providing a basis for further in-depth research on their subsequent functions.

### Effects of acousto-electric conversion fiber network–exosomes on the migratory capacity of BMSCs

**Fig. 5.**
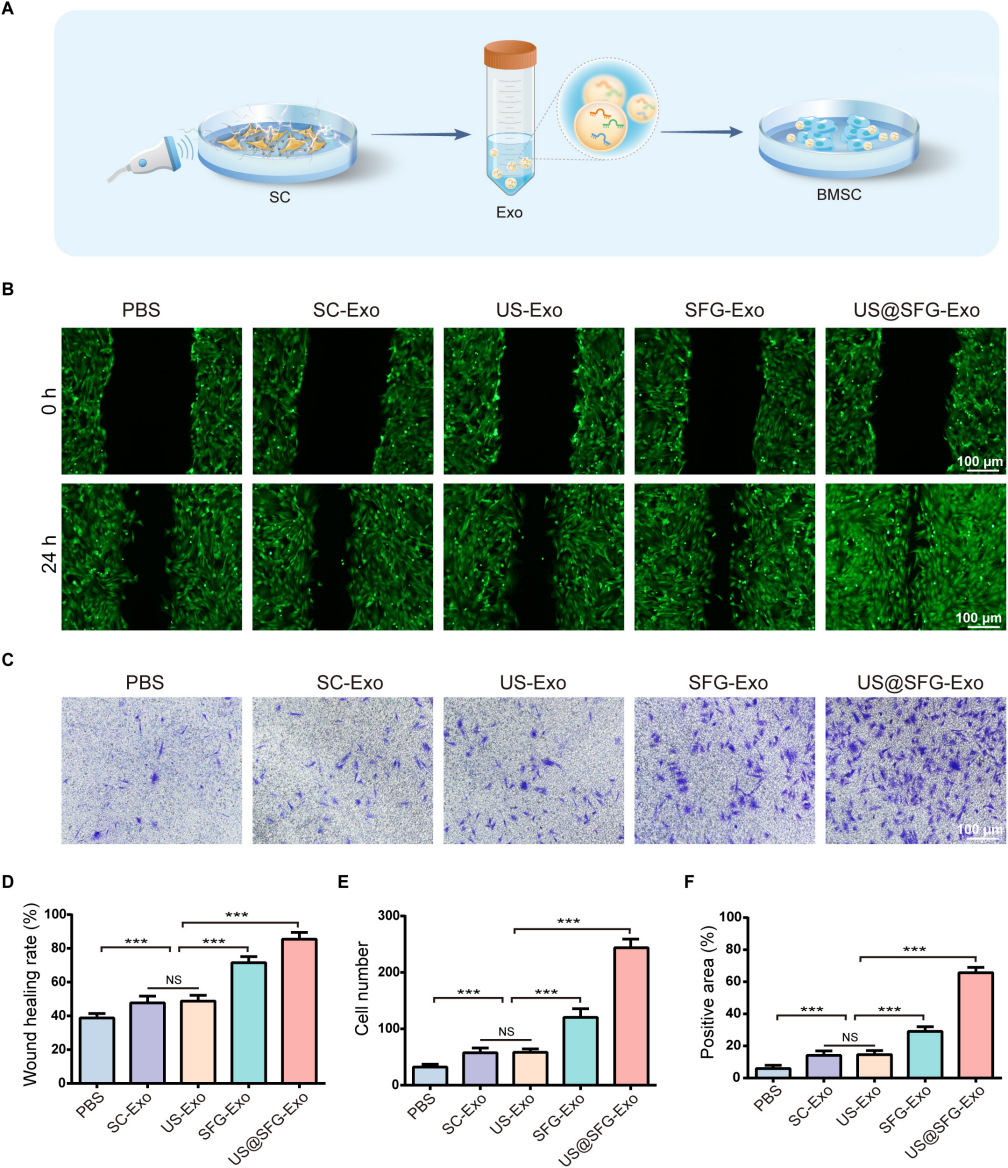
Analysis of the migration activity of SC exosomes induced by acousto-electric conversion fiber networks on BMSCs: (A) Schematic diagram of the model of BMSC migration in response to acousto-electric conversion fiber networks. (B) Scratch test showing BMSC migration with different exosome groups (PBS, SC-Exo, US-Exo, SFG-Exo, and US@SFG-Exo). (C) Transwell assay showing BMSC migration with different exosome groups (PBS, SC-Exo, US-Exo, SFG-Exo, and US@SFG-Exo). (D) Quantification of wound healing rates. (E) Quantification of the number of migrating cells. (F) Quantification of the positive area of migrating cells (NS, no significant difference; ***P < 0.05, *n* = 3).

The migratory capacity of BMSCs plays a pivotal role in bone tissue repair by directly determining stem cell recruitment efficiency to injury sites [25,26]. Exosomes from the SCs of each group were extracted and added to BMSC culture medium (Fig. 5A). The effects of the 4 types of exosomes on BMSC migration were evaluated using scratch and Transwell assays. As shown in Fig. 5B to F, after 24 h of incubation, the phosphate-buffered saline (PBS) group exhibited normal proliferation rates in both the scratch and Transwell assays. In contrast, the migration rates of the BMSCs cocultured with SC-Exos and US-Exos were faster than that of the PBS group. Moreover, the migration rates of the BMSCs cocultured with the SFG-Exos and US@SFG-Exos were significantly greater than those of the other 2 exosome groups, with the US@SFG-Exo group demonstrating the most substantial effect. This observed synergy is likely attributable to 2 mechanisms: (a) the dynamic electrical stimulation from the acousto-electric conversion fiber networks activates voltage-gated calcium channels, thereby promoting cytoskeletal reorganization; and (b) the topographical cues provided by the SFG matrix offer a favorable growth microenvironment for the migration of BMSCs. As a result, acousto-electric conversion fiber networks markedly enhance the migratory capacity of BMSCs, indicating that the acousto-electric microenvironment, in conjunction with exosome-mediated bioelectrical signals, may induce a cascading amplification effect.

### In vitro evaluation of the osteogenic potential of Schwann-derived exosomes

**Fig. 6.**
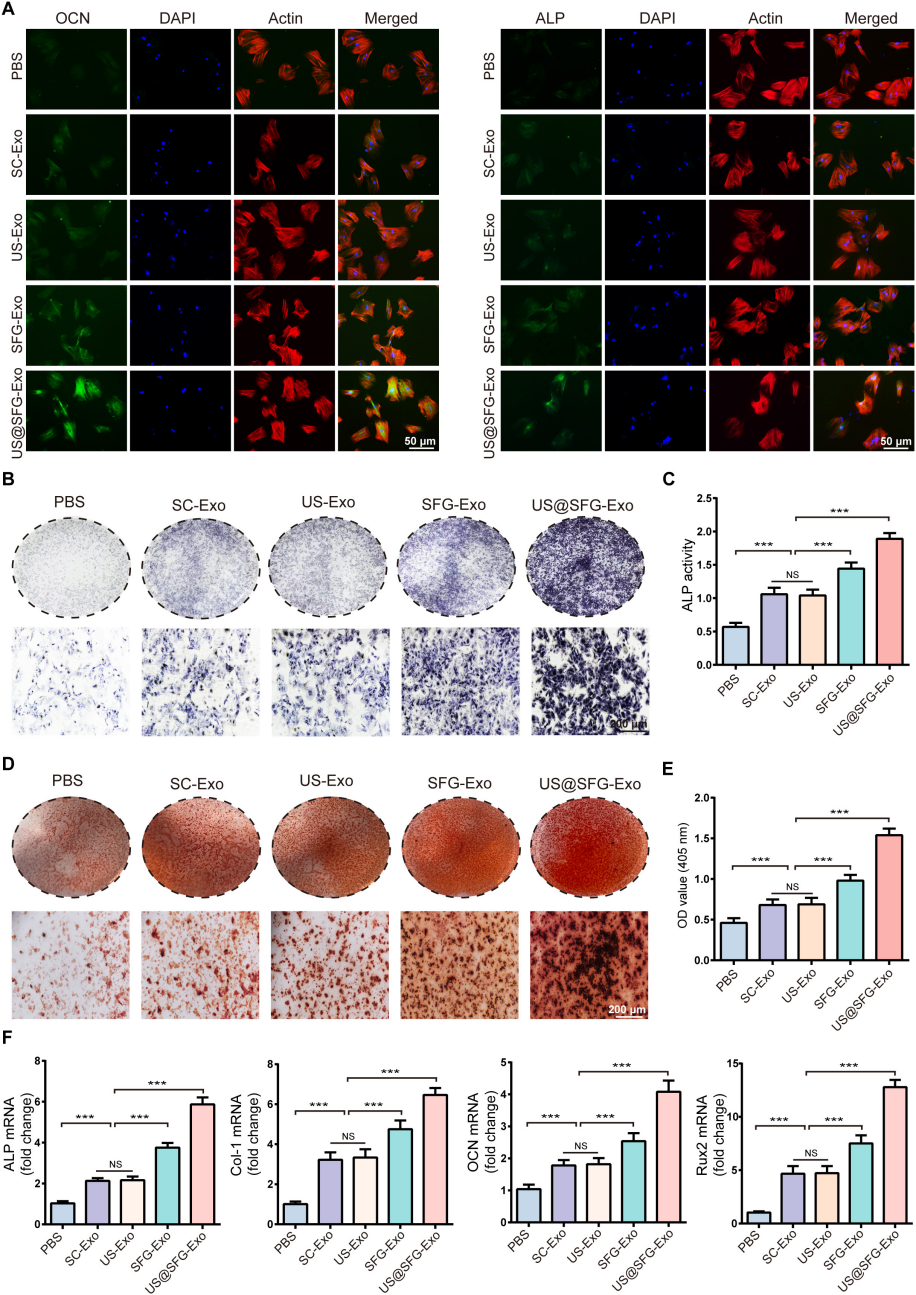
SC exosomes induced by acousto-electric conversion fiber networks promote BMSC bone differentiation in vitro: (A) Immunofluorescence staining of BMSCs. (B and C) Representative images and quantitative analysis of ALP staining. (D and E) Representative images and quantitative analysis of ARS staining. (F) RT-PCR analysis of osteogenic gene expression (NS, no significant difference; ***P < 0.05, *n* = 3).

During bone repair, the recruitment and subsequent osteogenic differentiation of BMSCs constitute essential biological processes in bone regeneration [27,28]. To evaluate the osteogenic potential of SC-derived exosomes induced by acousto-electric conversion fiber networks, we cocultured BMSCs with exosomes from different treatment groups (PBS, SC-Exo, US-Exo, SFG-Exo, and US@SFG-Exo) and systematically assessed their effects through immunofluorescence, alkaline phosphatase (ALP) staining, Alizarin Red S (ARS) staining, and reverse transcription polymerase chain reaction (RT-PCR) analyses. As shown in Fig. 6B and C, ALP staining revealed significantly greater staining intensity and activity in the US@SFG-Exo group than in the other groups, indicating that US@SFG-Exo effectively enhances the early osteogenic differentiation of BMSCs. ARS staining revealed a marked increase in calcium nodule formation in the US@SFG-Exo group, suggesting its superior ability to promote extracellular matrix mineralization (Fig. 6D and E). These findings were consistent with the immunofluorescence staining results for osteocalcin (OCN) and ALP (Fig. 6A), confirming that US@SFG-Exo not only accelerates osteogenic differentiation but also drives BMSCs into the mineralization maturation phase. Furthermore, RT-PCR analysis showed significantly elevated mRNA levels of ALP, Col-1, OCN, and Runx2 in the US@SFG-Exo group, further supporting its potent pro-osteogenic effects. These findings suggest that acousto-electric conversion fiber networks can enhance BMSC osteogenic differentiation through the modulation of SC-derived exosomes, thereby promoting neurogenic bone repair.

### High-throughput sequencing analysis

**Fig. 7.**
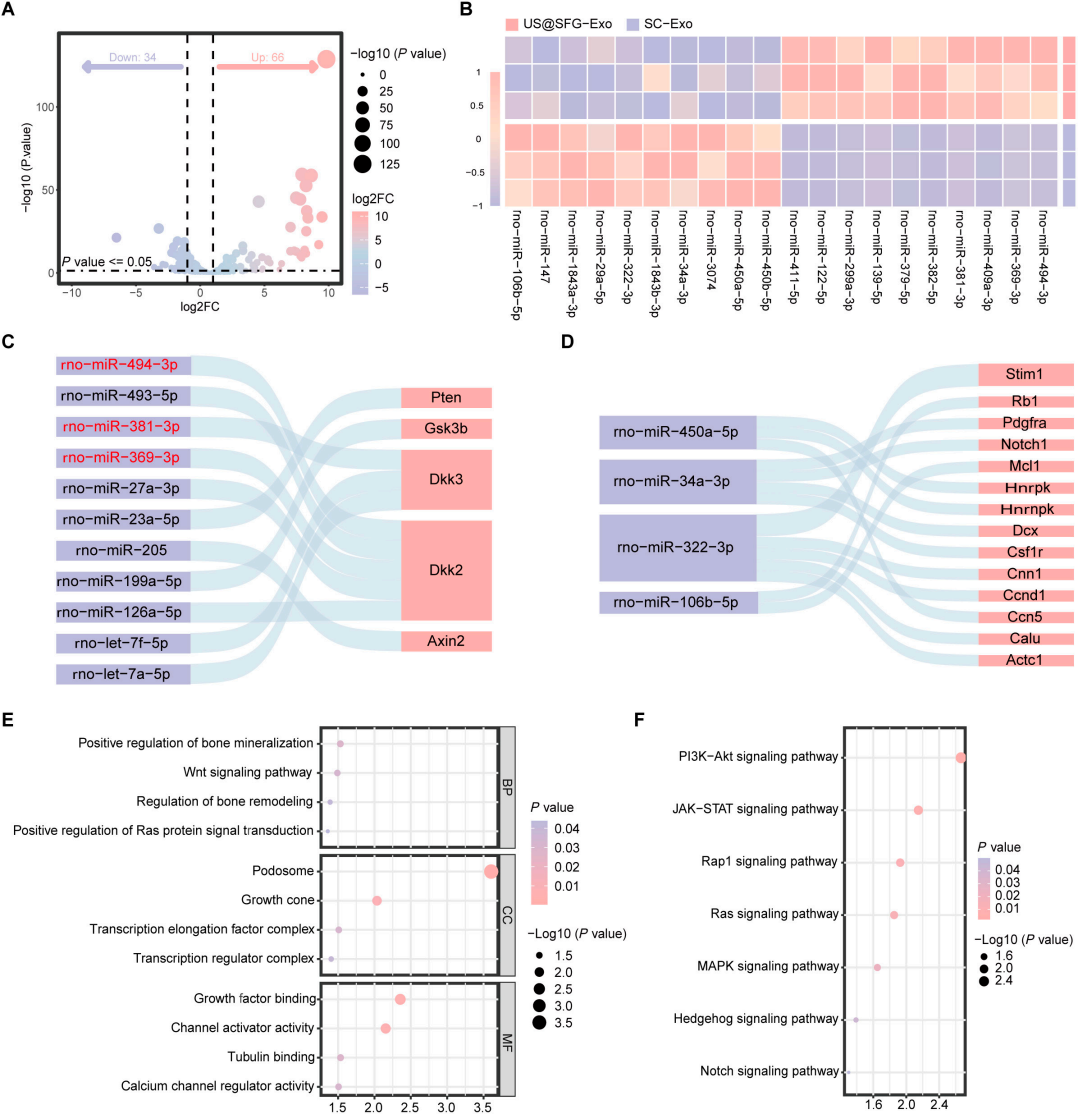
Functional analysis of genomic and signaling pathway changes induced by US@SFG-Exo: (A) Volcano plot of differential miRNA expression between SC-Exo and US@SFG-Exo, with purple and pink representing down-regulated and up-regulated miRNAs, respectively. (B) Heatmap showing the top 10 miRNAs with significant up-regulation and down-regulation. (C) Prediction of up-regulated miRNAs in US@SFG-Exo that directly target osteogenesis-related genes based on database analysis. (D) Identification of target genes for down-regulated miRNAs through database mining. (E and F) Gene Ontology and KEGG enrichment analysis of target genes of the top 10 down-regulated miRNAs.

High-throughput sequencing was utilized to examine the molecular process through which US@SFG-Exo aids in osteogenesis. A volcano plot was constructed, which revealed that, compared with the SC-derived exosome group, the US@SFG-Exo group contained 66 up-regulated and 34 down-regulated miRNAs (Fig. 7A). A heatmap was created to visualize the expression levels of the top 10 miRNAs with the greatest up-regulation and down-regulation (Fig. 7B). Based on predictions from the miRecords, miRTarBase, and TarBase databases, the target genes of both the up-regulated and down-regulated miRNAs in the US@SFG-Exo group could be identified. The results revealed that 11 up-regulated miRNAs directly target negative regulators of the PI3K/Akt and Wnt signaling pathways, including PTEN, GSK3β, DKK2, DKK3, and Axin2. Notably, miRNA-494-3p, miRNA-381-3p, and miRNA-369-3p were among the top 10 most significant miRNAs (Fig. 7C). Unfortunately, the down-regulated top 10 miRNAs were linked to genes that regulate the PI3K/Akt and Wnt pathways. These findings suggest that US@SFG-Exo may employ a dual-pronged strategy: up-regulating pro-repair miRNAs to activate key PI3K/Akt and Wnt signaling pathways, while down-regulating inhibitory miRNAs to release constraints on osteogenic pathways, thereby creating a synergistic multi-pathway effect (Fig. 7D). Subsequently, Gene Ontology enrichment analysis indicated that the target genes of the down-regulated miRNAs in US@SFG-Exo are involved in processes such as bone mineralization, bone remodeling, positive regulation of RAS protein signaling, and the Wnt signaling pathway (Fig. 7E). Kyoto Encyclopedia of Genes and Genomes (KEGG) analysis revealed that the miRNA–mRNA network regulated by US@SFG-Exo was enriched primarily in osteogenic-related signaling pathways, including the PI3K/Akt, JAK, RAS, Hedgehog, and Rap1 signaling pathways (Fig. 7F). These findings provide strong evidence that US@SFG-Exo activates the PI3K/Akt and Wnt pathways, either directly or indirectly, thereby enhancing bone repair.

### In vivo evaluation of the ability of US@SFG to promote osteogenic repair via high-throughput sequencing analysis

**Fig. 8.**
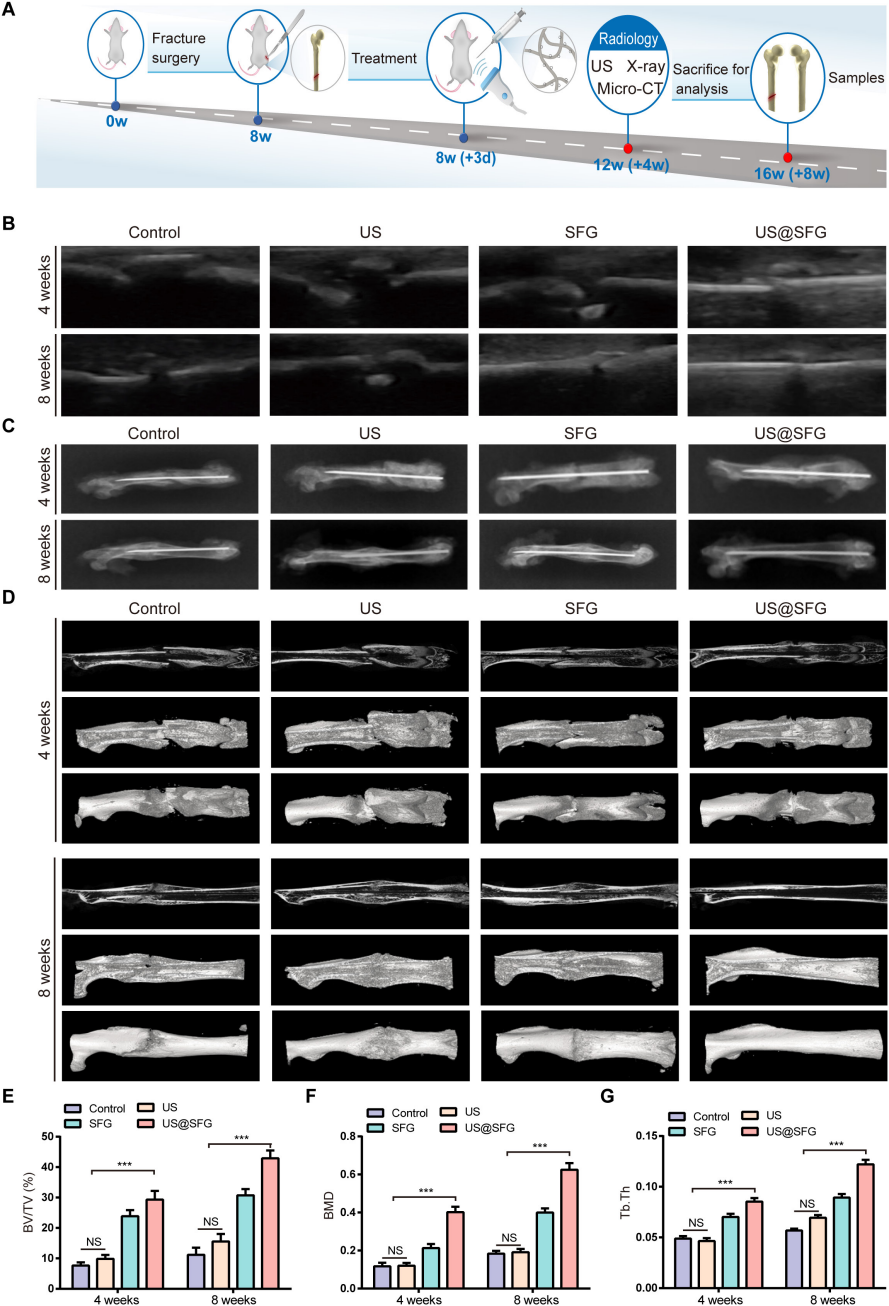
US@SFG promotes in vivo osteogenic repair: (A) Schematic diagram of diagnosis and treatment of femoral fracture in rats treated with different materials in each group. w, weeks; d, days. (B) Ultrasound imaging evaluation of femoral fracture repair at 4 and 8 weeks posttreatment. (C) X-ray images showing femoral fracture repair at 4 and 8 weeks for each treatment group. (D) 3D micro-CT reconstruction of femoral fractures at 4 and 8 weeks posttreatment. (E to G) Quantitative analysis of osteogenic indices, including BV/TV, BMD, and Tb.Th, reflecting bone volume and bone density at the fracture site (NS, no significant difference; ***P < 0.05, *n* = 3).

To assess the osteogenic repair effect of US@SFG comprehensively, both its neurorepair and osteogenic properties were evaluated in vivo. A rat femoral midshaft fracture model was established for this purpose. The control group included rats with fractures that were not treated, whereas the other groups were named US group, SFG group, and US@SFG group according to the different treatment methods. Eight weeks after implantation, major organ tissue samples were collected and analyzed histologically using hematoxylin and eosin (HE) staining (Fig. S4). No significant organ damage was observed in any of the treatment groups, indicating that the short fiber networks used in this study exhibited good biocompatibility, which was crucial for in vivo applications. To dynamically observe the effect of US@SFG on fracture healing, the femoral fracture healing of rats was conducted using ultrasound imaging, x-ray, and micro-CT at 4 and 8 weeks postimplantation (Fig. 8A). Ultrasound imaging revealed that US@SFG significantly promoted narrowing of the fracture gap, with callus formation starting from absent to present and the fracture line gradually disappearing, suggesting enhanced fracture healing (Fig. 8B). Similar results were observed in the x-ray images (Fig. 8C), demonstrating that the US@SFG accelerates early callus formation.

**Fig. 9.**
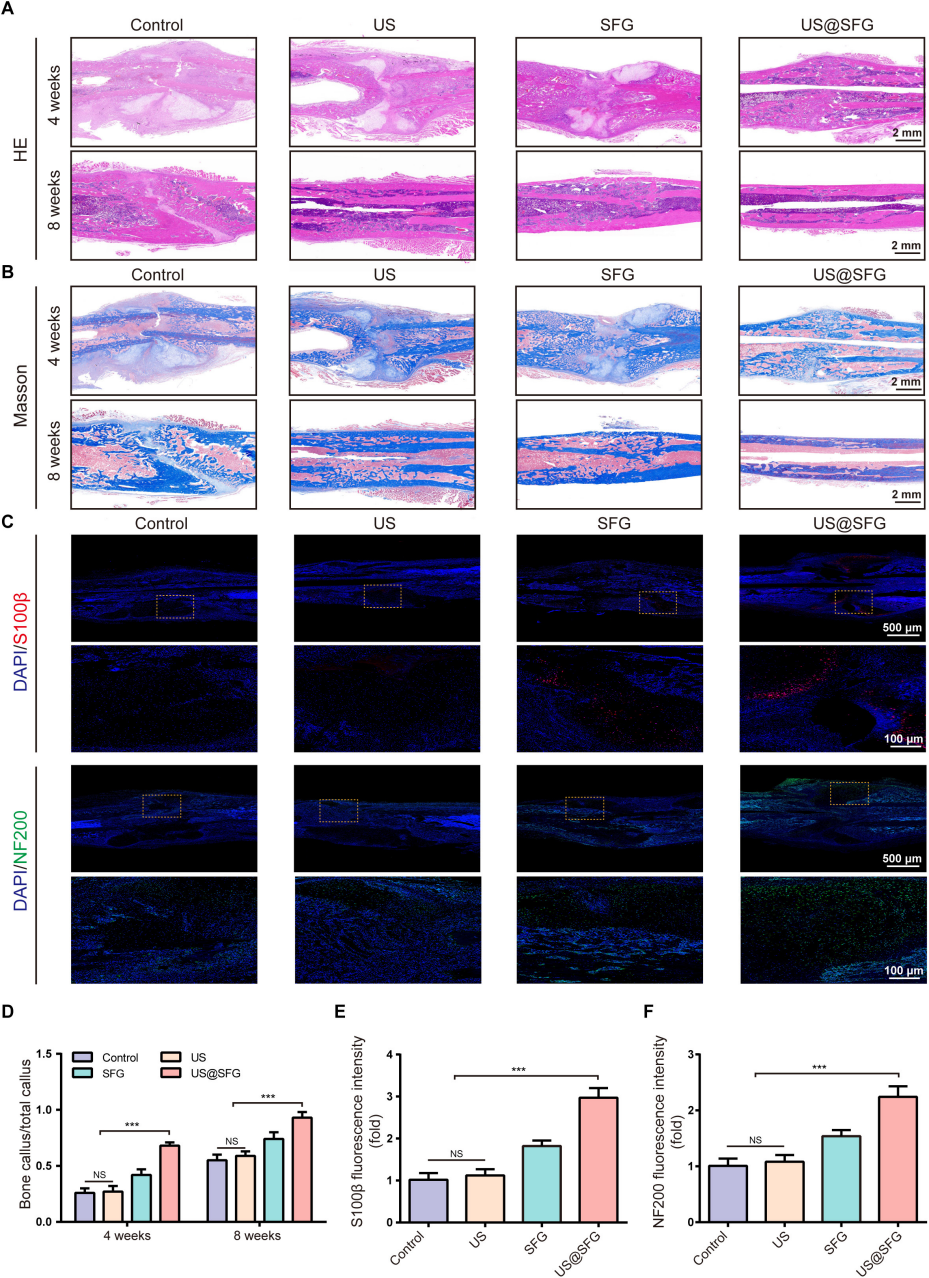
Histological analysis of bone tissue: (A) HE staining, (B) Masson staining, and (D) quantitative analysis of bone tissue at 4 and 8 weeks after treatment for each group. (C) Immunofluorescence staining and quantitative analysis (E and F) of S100β and NF200 of different materials in each group after 2 weeks of treatment (NS, no significant difference; ***P < 0.05, *n* = 3).

Bone tissue samples were gathered at 4 and 8 weeks postimplantation for the purposes of micro-CT 3-dimensional reconstruction and histological analysis, with the aim of evaluating local new bone formation. As shown in Fig. 8D, the US@SFG group presented significantly higher bone mineral density (BMD), bone volume-to-total volume ratio (BV/TV), and trabecular thickness (Tb.Th) compared to other groups, indicating not only enhanced bone mass but also improved microarchitecture (Fig. 8E to G). Notably, both the SFG and US@SFG groups exhibited superior osteogenic capacity over the control and US groups at 4 weeks, suggesting that the physical scaffolding provided by the fibrous matrix serves as the foundation for bone repair, whereas the combined ultrasound–electrical stimulation in the US@SFG group amplified this effect. Histological analysis (HE and Masson staining) at 4 weeks revealed abundant osteoblast accumulation and newly formed collagen fibers in the US@SFG group, whereas the control group primarily showed fibrous callus formation (Fig. 9A, B, and D). These findings correlated well with the radiographic observations, where evident fracture lines persisted in the control groups. By 8 weeks, the US@SFG group achieved complete fracture union with mature lamellar bone formation, indicating advanced healing stage. To further investigate the role of US@SFG in promoting neurogenic bone repair, immunofluorescence staining was conducted to examine the expression of neurorepair markers S100ꞵ and NF200 in fracture tissues at 2 weeks postoperation (Fig. 9C, E, and F). The US@SFG group exhibited the highest expression of S100ꞵ and NF200 at the fracture site, indicating enhanced neurorepair activity in the local tissue. As expected, the US@SFG group showed the most effective bone repair, likely due to the ability of acousto-electric conversion fiber networks to regulate nerve repair around the bone injury site, thereby promoting tissue regeneration and fracture healing.

### Transcriptomic analysis of US@SFG in neuro-bone repair

**Fig. 10.**
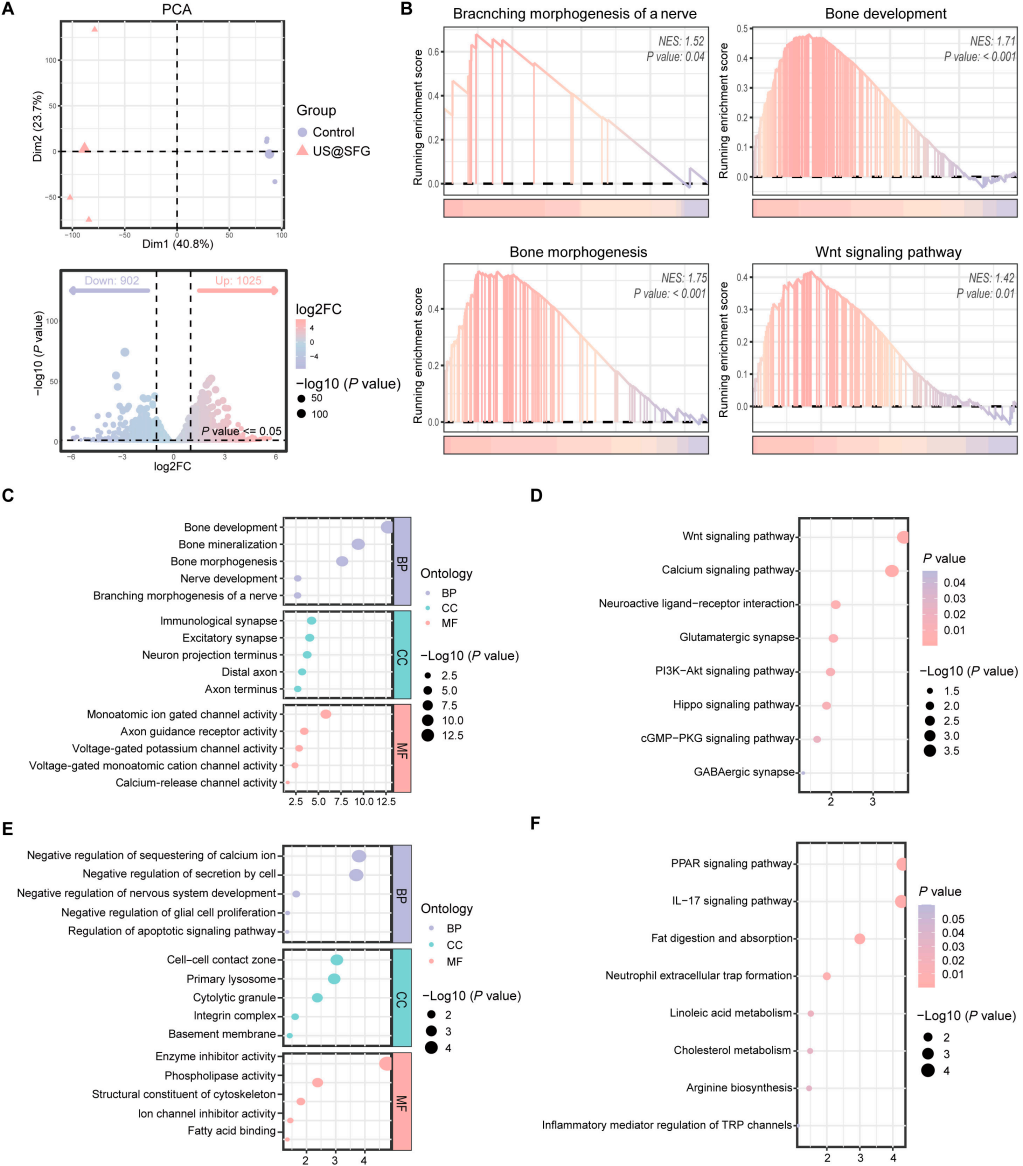
Transcriptomic analysis of bone tissue after implantation of US@SFG: (A) Volcano map of differential gene expression. (B) GSEA enrichment analysis of the overall pathway status of differentially expressed genes. (C and E) Gene Ontology enrichment analysis for functional annotation of differentially expressed genes. (D and F) KEGG analysis bubble plots showing differentially expressed gene enrichment pathway between the control group and the US@SFG group.

To further explore the neuro-bone repair effects and underlying mechanisms of acousto-electric conversion fiber networks at the fracture site, tissue samples from the fracture model and US@SFG groups were collected after 2 weeks of treatment in the rat femur fracture model for transcriptome sequencing analysis. As shown in Fig. 10A, the analysis of differences in gene expression between the 2 groups revealed that there were 1,025 up-regulated genes and 902 down-regulated genes in the US@SFG group (log2FC >1, *P* value < 0.05), indicating that US@SFG treatment markedly affected the biological function of the fracture tissue.

Gene set enrichment analysis (GSEA) revealed a marked activation of multiple neurogenic and osteogenic signaling pathways following US@SFG treatment. These pathways include those associated with bone development, osteogenesis, neural branching, and the Wnt signaling pathway (Fig. 10B). Additionally, we performed Gene Ontology analysis to assess the functional enrichment of genes with differential expression between the 2 groups, considering cellular components, biological processes, and molecular functions (Fig. 10C and E). The results showed that genes with increased expression were intimately connected to the processes of bone development, mineralization, osteogenesis, neurogenesis, and neural branching. Regarding biological processes, the genes with increased expression were connected to excitatory synaptic activity, neuronal projection terminals, and distal axon-related functions. Conversely, the genes with decreased expression were associated with the inhibition of cellular secretion, neural system development, glial cell proliferation, and apoptotic signaling pathways.

In terms of molecular functions, the up-regulated genes were involved in ion channel activity and other tissue repair-related functions. KEGG pathway analysis provided a visual representation of gene, metabolite, and pathway interactions. The results revealed that the up-regulated genes in the US@SFG group were chiefly associated with signaling pathways related to bone morphogenesis, including the Wnt, PI3K/Akt, and Hippo signaling pathways and secretory-related neuroactive ligand–receptor interaction pathways (Fig. 10D and F). According to existing research reports, the Wnt/β-catenin pathway not only is the core driving force of osteogenic differentiation, but also promotes neurogenesis and myelin repair [29,30]. The activation of the Hippo pathway effector factors YAP/TAZ has been confirmed to simultaneously enhance the osteogenic differentiation of mesenchymal stem cells and the myelin formation of SCs, which is highly consistent with the neuro-bone co-regulation phenomenon we observed [31,32]. Further analysis of the up-regulated genes of the PI3K/Akt pathway (such as mTOR and GSK3β) revealed that they might promote osteoblast protein synthesis through the mTORC1-P70S6K axis, and simultaneously amplify the Wnt signal through the Akt/GSK3β/β-catenin cascade to form a positive feedback regulatory loop. Based on these comprehensive findings, US@SFG markedly modulates both neural and bone repair processes, thereby accelerating bone regeneration.

## Conclusion

In this study, we innovatively developed an injectable acousto-electric conversion fiber network that can directly regulate the secretion of exosomal miRNAs from nerve SCs localized in injured bone tissue. Briefly, ultrasound-enhanced imaging functionality was successfully employed to precisely inject acousto-electric conversion fiber networks into the bone injury site. Subsequently, mechanical stimulation via ultrasound achieved precise acoustic and electrical transduction in tissues, and activated the PI3K/Akt and Wnt signaling pathways associated with osteogenesis in BMSCs by regulating the secretion of SC exosomal miRNA-494-3p, miRNA-381-3p, and miRNA-369-3p, which, in turn, promoted neuro-bone repair. In conclusion, this study is the first to develop injectable acousto-electric conversion fiber networks that regulate regionalized SCs in bone injury tissue for neuro-bone regeneration, providing new therapeutic strategies and targets for the interaction between bone and intrabone nerves.

## Materials and Methods

### Materials

PLA was provided by Daigang Biotech Co., Ltd. (Jinan, China). Gelatin, DA hydrochloride, hexafluoroisopropanol (HFIP), and tert-butanol were sourced from Aladdin Biochemical Technology Co., Ltd. (Shanghai, China). All other chemicals and reagents, unless specified, were acquired from Sigma-Aldrich. Fetal bovine serum (FBS), Dulbecco’s modified Eagle’s medium (DMEM), and penicillin/streptomycin were obtained from Gibco-Invitrogen. All consumables, including culture flasks, Transwell plates, 6-well plates, and 24-well plates, were purchased from Corning Inc.

### Synthesis of US@SFG

Since ꞵ-glycine is the most piezoelectric phase of glycine [33], glycine powder was dissolved in ultrapure distilled water and dripped onto clean Pt/Ti/SiO₂/Si wafers using a micropipette. The solution was then dried at room temperature until crystallization occurred. The collected crystals were ground using ceramic beads in a microtube ball mill [34]. Gelatin and PLA were dissolved in HFIP at a 4:1 weight ratio to prepare the gelatin/PLA spinning solution. The glycine crystals were integrated into the spinning solution and subjected to stirring for a duration of 6 h. Subsequently, the solution underwent electrospinning at a flow rate of 2 ml/h utilizing a 21-gauge blunt needle, with an applied electric field of 15 kV, to facilitate the collection of the short fiber membrane. The resulting 2-dimensional short fiber membrane was cut into pieces, which were then dispersed in tert-butanol and homogenized for 30 min at 13,000 rpm. Afterward, the samples were freeze-dried for 48 h to obtain uncrosslinked short fiber powder. To stabilize the structure, the scaffold was heated at 180 °C for 2 h to achieve thermal crosslinking, yielding stable short fibers. The crosslinked short fibers were homogenized and immersed in a GO solution (4 mg/ml). Finally, the GO-modified fibers were placed in a DA tris-buffer solution and reacted at 60 °C for 24 h to reduce the GO. The final product was washed with deionized water to obtain US@SFG.

### Characterization of US@SFG

The morphology of the prepared materials was examined using a digital camera (Canon, Japan) and a scanning electron microscope (FEI, USA). The chemical composition of the fiber networks was analyzed using XPS (Kratos Analytical, UK). To assess the piezoelectric properties of the US@SFG, one side of a flexible gold electrode was fixed at the bottom of a mold. The US@SFG was gently poured into the mold, and another gold electrode was placed on top of the solution. The piezoelectric output was measured using a piezoelectric testing system, which consisted of a general digital multimeter (Keithley-6514, USA) and a programmable linear motor connected to a computer. Each sample was subjected to low-intensity focused ultrasound. The piezoelectric conversion performance was tested under ultrasound parameters of 650 kHz frequency, 50% duty cycle, and 60 s exposure time. The piezoelectric output of the US@SFG was measured at ultrasound intensities of 0.5, 1, and 1.5 W/cm^2^ using an oscilloscope connected to an electrochemical workstation. The conductivity of the scaffold was evaluated by connecting a small light bulb to a closed-loop circuit to test the conductivity of US@SFG in both dry and wet states. Additionally, the electrical conductivity of the samples was conducted with a resistivity tester.

### Cell culture

BMSCs were extracted from SD rats. Briefly, muscle and connective tissue were removed from the femur and tibia of the rats [35]. After removing the bone ends, bone marrow was aspirated and cultured in DMEM supplemented with 10% FBS. SCs, provided by Shanghai Jiao Tong University, were also cultured in DMEM containing 10% FBS.

### Biocompatibility evaluation of US@SFG

The biocompatibility of the US@SFG was assessed using a combination of assays, including live/dead staining, cytoskeletal staining, CCK-8 proliferation assay, and LDH toxicity assay. For the CCK-8 and LDH assays, 96-well plates were used with 5,000 cells per well. The live/dead staining and cytoskeletal staining were conducted on 24-well plates, with 5 × 10^4^ cells per well. The cell viability was evaluated using a live/dead staining kit (Beyotime, China). LDH and CCK-8 assays were performed to assess cytotoxicity. The cytoskeletal staining was employed to assess the adhesion and morphology of SCs and BMSCs after treatment with the different material. After culturing for 1 and 3 days, cells were immobilized with 4% paraformaldehyde and then infiltrated with Triton X-100 for 5 min. Actin filaments were tagged using Alexa Fluor 594 phalloidin, and 4′,6-diamidino-2-phenylindole (DAPI) was employed to stain the nuclei. Cell morphology and the cytoskeleton were visualized under a fluorescence microscope (Nikon, Tokyo, Japan).

### Isolation and purification of SC-derived exosomes

Exosome isolation was performed using differential centrifugation as previously described [36]. Briefly, SCs were cultured to 80% confluence and treated with different materials. After an additional 48 h of incubation, the culture supernatant was collected from each group. Initially, cells were separated through centrifugation at 300×*g* for 10 min. Subsequently, larger molecular impurities were eliminated through centrifugation at 10,000×*g* for 30 min. The resulting supernatant was subsequently centrifuged at 100,000×*g* for 70 min to isolate the exosomes. The isolated exosomes were stored at −80 °C. The exosomes’ size distribution was assessed using nanoparticle tracking analysis (NTA), and their morphology and size were visualized and characterized through TEM. Western blotting was employed to analyze the expression levels of exosomal surface markers CD9, CD63, CD81, and TSG101, in addition to the negative exosomal marker Calnexin. To evaluate the uptake of exosomes by BMSCs, the exosomes were labeled with the red fluorescent dye PKH26, following the protocol provided by the manufacturer [37]. Subsequently, BMSCs were incubated with the labeled exosomes at 37 °C for durations of 4 and 8 h.

### In vitro assessment of BMSC migration

The primary methods used to evaluate the migration of BMSCs induced by SC-derived exosomes from US@SFG include the cell scratch test and the Transwell migration assay [38]. In the cell scratch assay, BMSCs were seeded at a density of 5 × 10^5^ cells per well. Wound scratches were created using the tip of a 10-μl pipette, and detached cells were subsequently removed by washing with PBS. The remaining cells were incubated in a medium supplemented with exosomes for 24 h. Prior to imaging, the cells were subjected to live/dead staining, and the wound areas were documented using fluorescence microscopy. In the Transwell migration assay, 2 × 10^4^ BMSCs were placed in the upper chamber of a Transwell plate, and a serum-free medium was used for cultivation. Simultaneously, the lower chamber was filled with culture medium containing exosomes secreted by SCs from various treatment groups. After 12 h of incubation, cells remaining on the upper chamber were removed, while those that had migrated were fixed with 4% paraformaldehyde, stained with crystal violet, and subsequently imaged.

### In vitro osteogenic potential assessment

BMSCs were seeded into 24-well culture plates and maintained in an osteogenic differentiation medium. This medium comprised exosome-depleted FBS, penicillin/streptomycin, dexamethasone, ascorbic acid, glutamine, and ꞵ-glycerophosphate. Following this, PBS, SC-Exo, US-Exo, SFG-Exo, or US@SFG-Exo was introduced to the corresponding groups. ALP staining (Beyotime, Shanghai, China) and activity assays were performed. The extent of ECM mineralization was evaluated using ARS staining (Beyotime, Shanghai, China). Additionally, the expressions of osteogenic proteins ALP and OCN were detected by immunofluorescence staining. Total RNA was extracted from the treated BMSCs, and the expression of osteogenic-related genes (Col-1, ALP, OCN, and Runx2) was quantified by RT-qPCR. The 2^−ΔΔCt^ method was employed to assess the relative levels of gene expression, with primer sequences available in the Supplementary Materials (Table S1).

### Establishment of animal models

All animal experiments received approval from the Animal Ethics Committee of the First Affiliated Hospital of Chongqing Medical University. The experimental subjects consisted of Sprague–Dawley rats, which were randomly allocated into 4 distinct groups: Control, US, SFG, and US@SFG. Anesthesia was administered to the rats through an intraperitoneal injection of 2% sodium pentobarbital in saline solution. After exposure of the femur, a fracture model was created using forceps at the midshaft of the femur. The fracture end was inserted with Kirschner needle for internal fixation, and then the wound was sutured in layers. SFG and US@SFG short fiber network materials were accurately injected into the fracture tissue under ultrasound imaging 3 days after operation. Ultrasound treatment was given to the US group and the US@SFG group, and all animals were injected with antibiotics for 3 days to prevent infection.

### Imaging observation

Rats from each group underwent imaging examination at 4 and 8 weeks after the surgery. First, ultrasound imaging was performed to assess fracture healing. Subsequently, the femurs were collected and preserved in 4% paraformaldehyde. X-ray and micro-CT scans were then conducted on the femurs. Three-dimensional images were reconstructed using CTvox software (Version 3.3.0, Bruker). Bone remodeling and regeneration were quantitatively assessed using parameters such as bone volume fraction (BV/TV), BMD, and Tb.Th.

### Histological evaluation

Bone tissue samples were collected from the animals in each group after 2 weeks of treatment. The specimen was preserved in 4% paraformaldehyde, followed by decalcification, embedding, slicing, and blocking with normal serum to avoid nonspecific binding. The sections were then incubated with S100ꞵ and NF200 antibodies (1:100 dilution) for 24 h, followed by incubation with a secondary antibody conjugated to a specific fluorescent label. The nucleus was stained using DAPI, and the results were observed through a fluorescence microscope. Moreover, HE and Masson staining were performed on tissue sections at 4 and 8 weeks postimplantation to evaluate femoral fracture healing.

### Statistical analysis

All statistical analyses were performed using GraphPad Prism 9.0 software. Data are presented as means ± standard deviations (SDs). For comparisons between 2 groups, Student’s *t* -test was used. For comparisons among multiple groups, one-way analysis of variance (ANOVA) was employed. A significance level of *P* < 0.05 was considered statistically significant.

## Data Availability

All data included in this study are available upon request by contact with the corresponding authors.
