# Supplementary material for: Acousto-Electric Conversion Fiber Networks via Regional Activation of Schwann Cell-Derived Exosomes for Neurogenic Bone Regeneration
Source: Research (Wash D C). 2025 Jul 15;8:0769. doi: 10.34133/research.0769 (PMC12260224; doi:10.34133/research.0769)
Supplement: Supplementary 1 — Figs. S1 to S4 Table S1 [file research.0769.f1.doc]

**Supplementary Materials**

**Acousto-electric Conversion Fiber Networks via Regional Activation of Schwann Cell-Derived Exosomes for Neuro–bone Regeneration**

Weiwei Yi^1†^, Xiaoyu Han^1†^, Fan Wang^2†^, Qiuyu Tang^1^, Huzhe Liu^3^, Bo Liao^1^, Jieliang Shen^4^, Juan Wang^2*^, Wenguo Cui^1,2*^, Dingqun Bai^1*^

^1.^Department of Rehabilitation Medicine, Key Laboratory of Physical Medicine and Precision Rehabilitation of Chongqing Municipal Health Commission, The First Affiliated Hospital of Chongqing Medical University, No.1 Youyi Road, Yuzhong District, Chongqing, 400016, China.

^2^. Department of Orthopaedics, Shanghai Key Laboratory for Prevention and Treatment of Bone and Joint Diseases, Shanghai Institute of Traumatology and Orthopaedics Ruijin Hospital Shanghai Jiao Tong University School of Medicine, Shanghai 200025, China.

^3^. Department of Orthopaedics, People's Hospital of Chongqing Liang Jiang New Area, Chongqing 400016, China.

^4^. Department of Rehabilitation Medicine, Bishan Hospital of Chongqing Medical University, Bishan Hospital of Chongqing, Chongqing 402760, China.

^†^ These authors contributed equally to this work.

^*^Corresponding authors: Juan Wang, juanwang1006@126.com; Wenguo Cui, [wgcui@sjtu.edu.cn](mailto:wgcui@sjtu.edu.cn) and Dingqun Bai, baidingqun @hospital.cqmu.edu.cn.


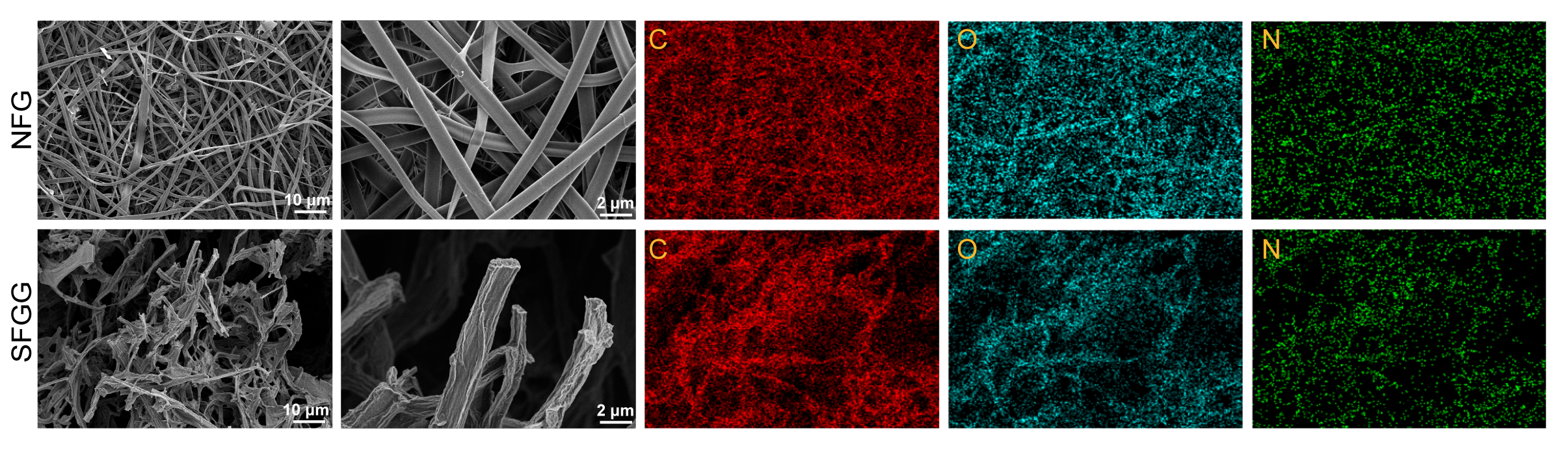


**Figure S1.** Scanning electron microscope observation of glycine-containing short fiber film (NFG) and GO oxidized short fiber (SFGG).


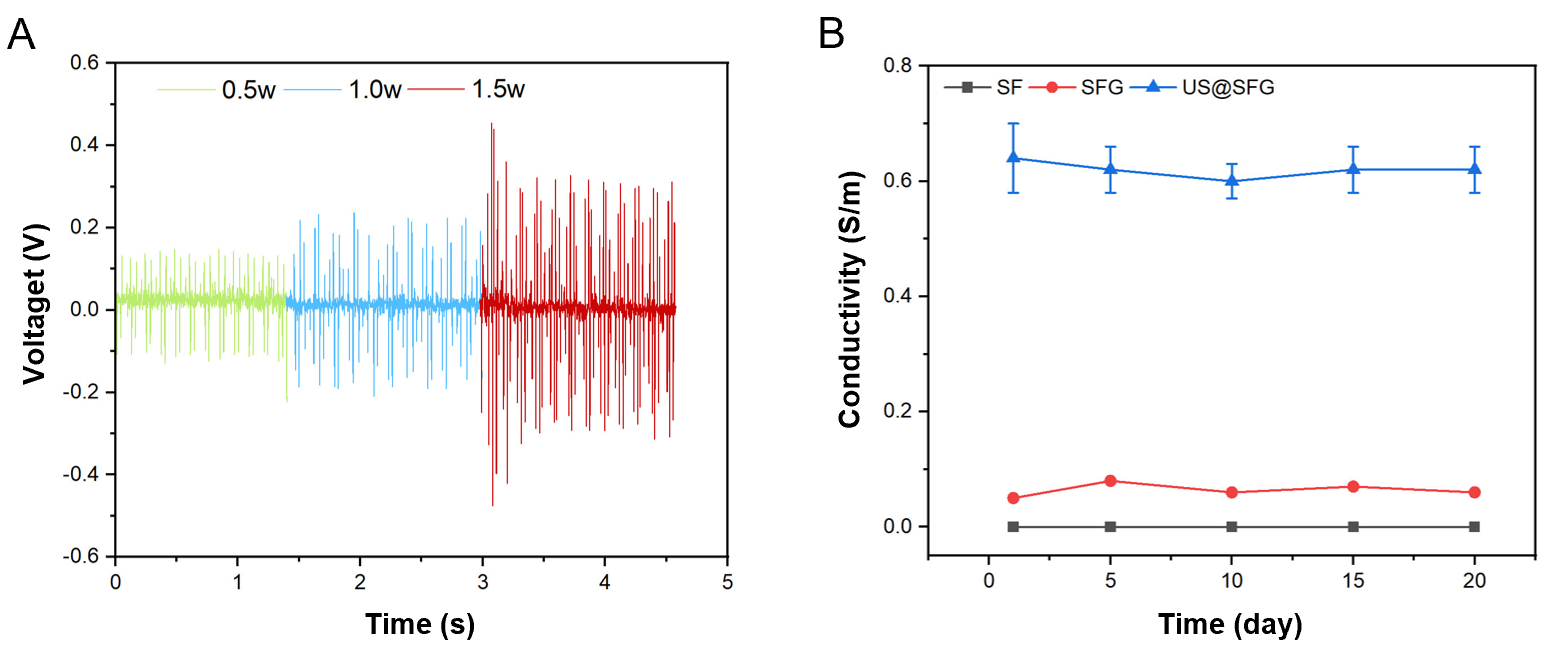


**Figure S2.** Electrical stability test and piezoelectric properties of short fibers: (**A**) Electrical stability testing of electroactive short fibers. (**B**) Differences in piezoelectric properties between conventional short fibers and electroactive short fibers.


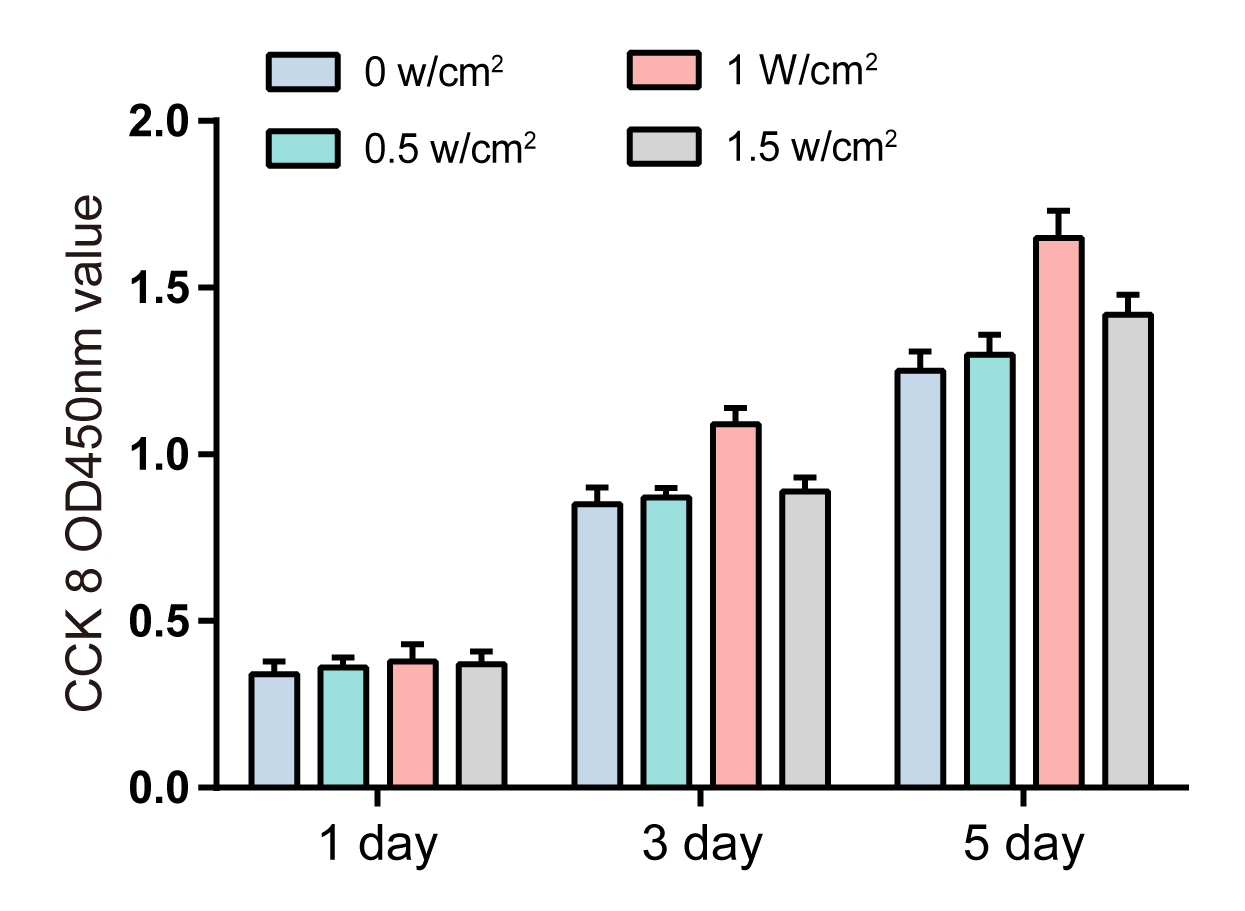


**Figure S3.** Cell viability of SCs induced by electroactive short fibers stimulated by different intensities of ultrasound measured by CCK-8 assay.


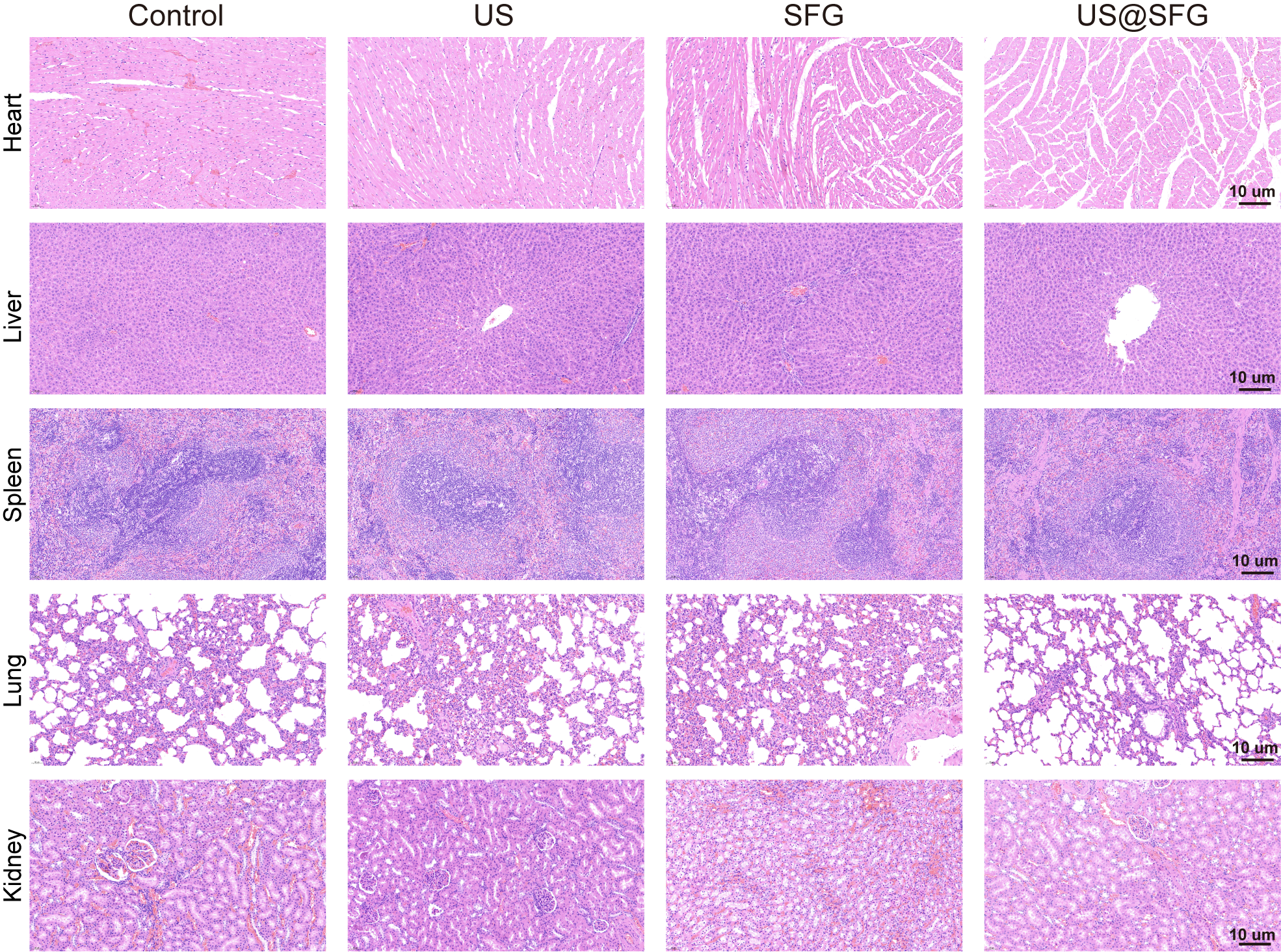


**Figure S4.** HE staining of organ tissue sections for the toxicity of samples on heart, liver, spleen, lung, and kidney.

**Table S1.** **Primers sequences used for RT-PCR assay**.

|  | **Primer** | **Forward (5'-3')** | | **Reverse (5'-3')** |
| --- | --- | --- | --- | --- |
|  | *Runx2* | | GGAACCAAGAAGGCACAGACAG | | --- | | | TGTCTGCCTGGGATCTGTAATCT | | --- | | |
|  | *Col-1* | AGCTCGATACACAATGGCCT | CCTATGACTTCTGCGTCTGG | |
|  | *OCN* | \| GAGCTCAACCCCAATTGTGAC \| \| --- \| | \| AACGGTGGTGCCATAGATGC \| \| --- \| | |
|  | *Gapdh* | \| CTGGAGAAACCTGCCAAGTATG \| \| --- \| | \| GGTGGAAGAATGGGAGTTGCT \| \| --- \| | |
